# A Potential Case of Acamprosate-Induced Syncope in an Elderly Patient: A Case Report and Literature Review

**DOI:** 10.7759/cureus.99283

**Published:** 2025-12-15

**Authors:** Khaled Al Khodari, Hong Hieu Troung, Diana Keratishvili, Laith Sorour, Tasneem Anagreh, Mouaz Al Khodari

**Affiliations:** 1 Internal Medicine, Saint Francis Hospital, Evanston, USA; 2 Internal Medicine, Syrian Private University, Damascus, SYR

**Keywords:** acamprosate, adverse drug reaction, alcohol use disorder, side effects of medical treatment, syncope

## Abstract

Acamprosate is commonly prescribed to maintain abstinence in patients with alcohol use disorder (AUD) and is generally well tolerated; however, syncope is not typically recognized as a potential adverse effect. We present the case of an elderly woman with no prior cardiac or neurological history who developed unexplained syncope shortly after initiating acamprosate. A comprehensive diagnostic workup was unremarkable. Acamprosate was discontinued, and no further episodes occurred during follow-up. Although a definitive causal relationship cannot be confirmed, the absence of other identifiable causes and the temporal association raise the possibility of a link between acamprosate and syncope. This case underscores a potentially unrecognized adverse effect of acamprosate, particularly relevant in elderly patients, and emphasizes the need for clinicians to be aware of this possible association.

## Introduction

Acamprosate is an FDA-approved medication for the treatment of alcohol use disorder (AUD). When combined with psychosocial support, it aids in maintaining abstinence. It has also demonstrated effectiveness in helping individuals regain abstinence following a relapse, with some evidence suggesting it reduces both the frequency and quantity of alcohol consumption during such episodes [1]. A meta-analysis of 17 studies involving 4,087 participants concluded that acamprosate was significantly more effective than placebo in maintaining abstinence at six months [2].

Acamprosate has a well-established safety profile. It is primarily eliminated unchanged through the kidneys and does not undergo hepatic metabolism. Data from 13 clinical trials indicate that it is safe, well tolerated, and associated with minimal drug interactions. The most commonly reported adverse effect is diarrhea, typically mild to moderate in severity and self-limiting. Other frequently observed side effects include fatigue, itching, abdominal discomfort, relapse to alcohol use, depression, headache, infections, and insomnia, most of which tend to resolve over time [3].

Severe neurological or cardiovascular side effects are rarely reported. However, extrapyramidal symptoms, including bradykinesia, muscle rigidity, slowed speech, and reduced arm swing, have been observed in two patients following initiation of the medication [4,5]. In another case, multifocal jerks were noted in the lower limbs of a young patient shortly after initiating acamprosate. The frequency of these jerks increased following a dose escalation [6]. All symptoms resolved entirely after acamprosate was discontinued.

Syncope is defined as a sudden and reversible loss of consciousness caused by a temporary reduction in cerebral perfusion. It is a relatively common condition, affecting about one-third to one-half of the general population at some point in their lives, with neurocardiogenic (vasovagal) syncope being the most frequent subtype. This occurs due to vagal activation, leading to hypotension and bradycardia. Other forms of reflex syncope include orthostatic hypotension, carotid sinus hypersensitivity, and situational syncope such as that triggered by urination or coughing. Cardiac causes include arrhythmias and structural abnormalities like hypertrophic obstructive cardiomyopathy or severe aortic stenosis. Less commonly, syncope may result from neurological conditions involving autonomic dysfunction, like stroke and neurodegenerative diseases [7-9].

To the best of our knowledge, syncope has not previously been reported as a potential adverse effect of acamprosate. In the case that follows, we hypothesize that acamprosate may have contributed to a syncopal episode through central nervous system dysregulation affecting autonomic control. It adds to the limited body of literature on acamprosate’s neurological safety profile and underscores the importance of clinical vigilance, particularly when prescribing this medication to elderly patients or those with potential autonomic vulnerability.

## Case presentation

A 77-year-old woman with a past medical history of breast cancer, hypothyroidism, major depression, and AUD, reporting daily consumption of a couple of glasses of alcohol for a long time, was admitted to the hospital after experiencing two episodes of syncope 10 days after being started on acamprosate 666 mg three times daily by her primary care physician (PCP). On the day of admission, she reported entering the kitchen in the evening when she suddenly felt profound weakness while standing at the counter. She lowered herself to the floor and experienced a brief loss of consciousness lasting a few seconds. Upon regaining consciousness, she sat up but soon experienced a recurrence of the same symptoms and another transient syncopal episode. Prior to these events, she noted generalized weakness. She denied any chest pain, shortness of breath, palpitations, headache, nausea, vomiting, or visual changes. There were no involuntary movements, urinary or fecal incontinence, or tongue biting. She did not sustain any head trauma. Following the episodes, she remained conscious and oriented but continued to feel weak, without any focal neurological deficits or recurrent symptoms. Apart from acamprosate, she has been taking levothyroxine 50 micrograms daily and venlafaxine 75 mg daily for a long time. She does not have a previous withdrawal history.

She was vitally stable during hospitalization, and her physical examination was completely normal. Aside from mild hypokalemia, with a potassium level of 3 mmol/L below the normal range of 3.5-5.5 mmol/L, the remainder of her laboratory workup, including complete blood count, liver, renal, and thyroid function tests, and other electrolytes, was within normal limits. Hypokalemia was treated with oral and intravenous potassium chloride.

**Figure 1:**
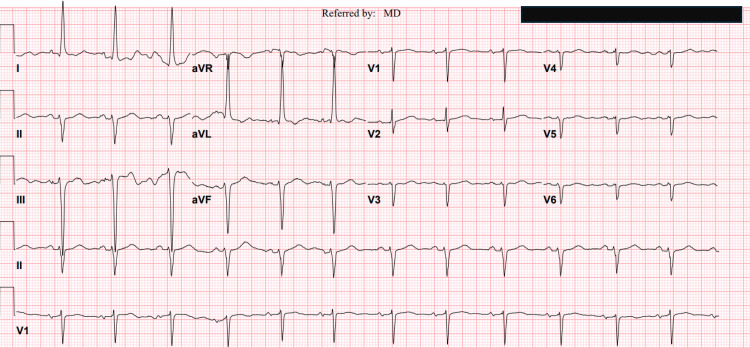
Electrocardiography (ECG) Electrocardiography (ECG) shows a sinus rhythm with a heart rate of around 75 beats per minute. Left ventricular hypertrophy (LVH) with a possible strain pattern and resultant left-axis deviation. There is no significant ST-segment deviation or arrhythmias. LVH, by itself, does not explain the syncope for this patient.

**Figure 2:**
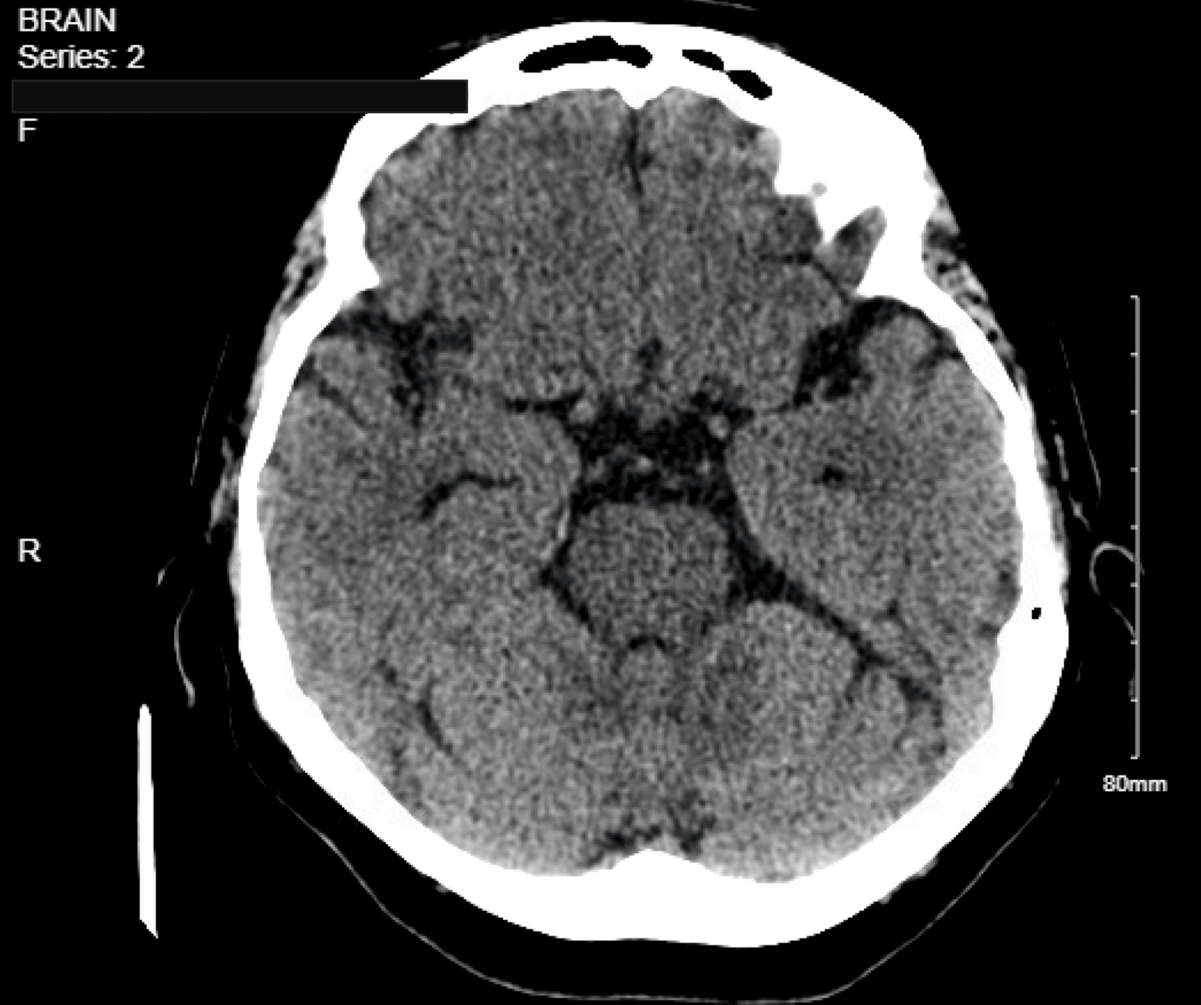
Head Computed Tomography (CT) Scan A non-contrast axial computed tomography (CT) scan of the brain demonstrates normal cerebral parenchymal attenuation without evidence of acute intracranial hemorrhage, mass effect, or midline shift. The ventricular system and cortical sulci appear within normal limits for the patient’s age. The basal cisterns are patent. No areas of abnormal hypoattenuation are identified to suggest acute ischemic infarction. The visualized portions of the posterior fossa, including the cerebellum and brainstem, are unremarkable. These findings ruled out acute brain insult that could have explained the syncopal attack.

The top differential diagnoses included vasovagal syncope, cardiac arrhythmia, structural heart disease such as severe aortic stenosis or acute myocardial infarction, and acute cerebrovascular events like stroke. However, the absence of typical prodromal symptoms, the occurrence of the second episode while the patient was seated, and the lack of chest pain, palpitations, or focal neurological deficits, combined with a normal syncope workup including orthostatic blood pressure measurements, electrocardiogram (ECG), echocardiography, two sets of cardiac troponin (4 > 5 pg/ml, normal less than 12 pg/ml), head computed tomography (CT) scan, and 48-hour telemetry monitoring, effectively ruled out these potential causes (Figure 1, 2). Hypokalemia is considered an unlikely contributing factor, given its mild severity, absence of associated ECG or telemetry abnormalities, and successful correction during hospitalization. Acamprosate was discontinued, and the patient remained clinically stable throughout her hospitalization without the need for additional interventions. At her one-month follow-up, she reported no recurrence of syncope or presyncopal symptoms. She continued to abstain from alcohol and remained under regular follow-up with her primary care physician and addiction specialist. Acamprosate was not reintroduced. Table 1 summarizes the key clinical events from the initiation of acamprosate to the patient’s syncope episode and subsequent follow-up.

**Table 1:** Timeline of Clinical Events Related to Acamprosate Use and Syncope The timeline table outlines the sequence of clinical events including the start of acamprosate therapy, onset of symptoms, syncope episode, emergency evaluation, discontinuation of the medication, and follow-up outcomes. Day 0 represents the date acamprosate was initiated.

Time Point	Event/Intervention	Clinical Details
Day 0	Acamprosate initiated	Started at 666 mg three times daily.Baseline vitals and labs normal. No prior history of syncope.
Day 10	Syncope at home	Collapsed at home. Brief loss of consciousness (<1 min). No seizure activity. ED vitals stable; ECG normal; labs unremarkable apart from mild hypokalemia.
Day 11	Admission under medicine	Cardiac enzymes normal, potassium replaced, imaging unremarkable. Acamprosate suspected as a possible cause. Medication discontinued.
48–72 hours post-discontinuation	Symptom resolution	No recurrent syncope. Completely asymptomatic.
1-month follow-up	Office visit	No recurrence of syncope. Patient stable off acamprosate. No alternative explanation identified.

## Discussion

Acamprosate, along with naltrexone, is recommended by the American Psychiatric Association (APA) as a first-line treatment for individuals with moderate-to-severe AUD who prefer pharmacologic therapy and are motivated to reduce alcohol consumption and achieve abstinence [10]. Although the exact mechanism of action of acamprosate remains unclear, it is thought to exert its therapeutic effects by modulating glutamatergic and GABAergic neurotransmission, thereby helping to stabilize the neurochemical imbalances associated with chronic alcohol use [11]. Serious adverse effects associated with acamprosate are rare, with the most frequently reported neurological side effects including headache, dizziness, insomnia, and irritability [12]. Syncope, however, is not a commonly recognized adverse effect. A review of clinical trials and post-marketing surveillance data has not demonstrated a definitive association between acamprosate and syncopal episodes.

As far as we are aware, this is the first reported case suggesting a potential association between acamprosate and syncope. While definitive causality cannot be established, the close temporal correlation between the onset of symptoms and the initiation of acamprosate, combined with the absence of an alternative identifiable cause and the resolution of syncope following discontinuation, raises the possibility of a causal relationship.

Although acamprosate was the most recently initiated medication, it is important to consider the potential contribution of the patient’s chronic medications. She had been on stable doses of venlafaxine and levothyroxine for the treatment of depression and hypothyroidism, respectively. Venlafaxine, a serotonin-norepinephrine reuptake inhibitor (SNRI), has been infrequently associated with autonomic dysfunction and orthostatic hypotension, typically at higher doses exceeding 225 mg/day [13,14]. In this case, the patient had been taking a low, unchanged dose of venlafaxine for an extended period, making it an unlikely contributor to her symptoms, particularly given that the medication was continued after discharge without recurrence of syncope.

Interestingly, there are some data supporting the use of SNRIs, including venlafaxine, in the treatment of refractory orthostatic hypotension and vasovagal syncope. This therapeutic effect is thought to result from the downregulation of postsynaptic serotonin receptor density, which may blunt the exaggerated serotonergic-mediated sympathetic withdrawal that contributes to hemodynamic instability in hypersensitive states [15]. Levothyroxine has been associated with cardiac arrhythmias, which may increase the risk of syncope. However, the patient’s thyroid function tests were within normal limits during hospitalization, and she had been maintained on a stable dose for a prolonged period. These factors make it unlikely that levothyroxine contributed to the syncopal episodes in this case.

As highlighted in the background, syncope is a multifactorial condition that may arise from neurocardiogenic, cardiac, or neurologic mechanisms, and it has not previously been reported as an adverse effect of acamprosate. The close temporal relationship between symptom onset and acamprosate initiation raises the question of a potential causal link. The exact mechanism of potential acamprosate-induced syncope is unclear. The observed symptom may reflect central nervous system dysregulation induced by acamprosate, particularly in elderly patients, which could impair blood pressure regulation and cerebral perfusion. Moreover, individuals with AUD often have reduced oral intake and excessive diuresis, predisposing them to dehydration and electrolyte imbalances, factors that may further contribute to the risk of syncope [16].

This case underscores the importance of clinical vigilance regarding rare or underrecognized adverse effects, particularly in populations with altered drug metabolism, increased sensitivity to central and autonomic changes, or polypharmacy. Greater awareness may aid in early detection and prevent recurrence in susceptible individuals.

From the patient’s perspective, the syncopal episode was unexpected and distressing. She reported that the event made her concerned about continuing the medication and about the possibility of recurrence. After learning that acamprosate may have contributed to the episode and that it would be discontinued, she expressed relief and felt reassured by the clear explanation and follow-up plan.

## Conclusions

This case draws attention to syncope as a potentially unrecognized adverse effect of acamprosate. Although a definitive causal relationship cannot be confirmed, temporal association and symptom resolution following discontinuation raise clinical suspicion. Clinicians may consider this potential relationship when evaluating unexplained syncope in patients receiving acamprosate. Continued pharmacovigilance and additional case reporting are needed to clarify whether this association is coincidental or clinically meaningful.
